# Anomalous acoustic phonons as the physical mechanism behind the adiabatic barocaloric effect on graphene

**DOI:** 10.1038/s41598-018-36525-7

**Published:** 2019-01-18

**Authors:** Ning Ma, Mario S. Reis

**Affiliations:** 1 Department of Physics, Taiyuan University of Technology, Taiyuan, 030024 China; 2 Department of Applied Physics, MOE Key Laboratory for Nonequilibrium Synthesis and Modulation of Condensed Matter, Xi’an Jiaotong University, Xi’an, 710049 China; 3 Institute of Physics, Fluminense Federal University, Av. Gal. Milton Tavares de Souza s/n, 24210-346 Niterói-RJ, Brazil

## Abstract

A graphene sheet is able to either heat up or cool down due to a mechanical strain: this is the adiabatic *barocaloric effect*. In order to understand the physical mechanism behind this effect, we have explored the adiabatic temperature change of the graphene and, for this purpose, we considered two contributions to the total entropy: a lattice entropy (depending on the transversal, longitudinal and anomalous out-of-plane acoustic phonons) and a strain entropy. We found that the adiabatic barocaloric effect only depends on the strain energy and the anomalous acoustic phonons, without terms due to the transversal and longitudinal acoustic phonons.

## Introduction

Caloric effects have been attracting the attention of the scientific community for the past decades because of the broad appealing for application in thermal devices^1^. Across the years, the concept has been expanded beyond the magnetocaloric borders and, nowadays, as popular as the former, other caloric effects are *in vogue*, as such as the barocaloric and the electrocaloric effects^2,3^. These effects can work even better when are present into a single material and, for this case, it is named as multicaloric effect^4^.

On the other hand, it is known that a strain on graphene mimics a pseudo-magnetic field, as theoretically described in references^5–7^, and experimentally verified by Levy and co-workers^8^. In this direction, in a similar fashion as we have done in our previous contribution studying the isothermal barocaloric effect on graphene^3^, strain produce a pseudo-magnetic field that, on its turn, produces a caloric effect.

The goal of the present effort is thus to understand the physical mechanism behind the barocaloric effect on graphene and, to achieve this goal, we have considered two contributions to the total entropy: one from the lattice and other from the strain. The former has two contributions from the acoustic phonons with a linear dependency with the phonons wavenumber *q* and one contribution with a quadratic *q*-dependency. This anomaly is responsible to rule the barocaloric effect on graphene and this finding is further explored along the text.

## Results

### Lattice entropy

The lattice entropy for a single layer graphene depends on three acoustic phonon modes. For phonons, their interaction corresponds (in the classical picture), to anharmonic vibrations of the atoms in the lattice. However, these vibrations in solids are, in practice, always weak; or, to say, ‘almost harmonic’. Therefore, to write these dependence, we start from the Helmholtz free energy of phonons in the harmonic approximation^9–11^:

1 $$F(T)={k}_{B}T\,\sum _{j=1}^{3N\nu }\,\mathrm{ln}\,(1-{e}^{-\hslash {\omega }_{j}/{k}_{B}T})+{\varepsilon }_{0}.$$

In this form, the free energy can be regarded as a superposition of independent harmonic oscillators, or normal mode of vibrations. Specifically, the first term is the standard temperature dependent bosonic contribution, with the summation running over *j* up to the number of normal mode of vibration (i.e., the vibrational degrees of freedom) 3 *Nν*, where *N* is the absolute number of crystallographic sub-lattices *A* and *B* in the lattice, *ν* = 2 is the number of atoms into those sub-lattices and the factor 3 is the possible space directions of vibration. Such a system with 3 *Nν* vibrational degrees of freedom may be regarded as an assembly of 3 *Nν* independent harmonic oscillators, each corresponding to one normal mode of vibration. The second term is the zero-temperature contribution and represents the energy of zero-point vibration of all atoms ${\varepsilon }_{0}={\sum }_{j=1}^{3N\nu }\,\hslash {\omega }_{j}/2$. This term is closely related to the number of atoms in the specimen and is temperature-independent; thereby, leading to a null contribution to the lattice entropy - this idea is only valid for the harmonic limit we are considering.

The ballistic thermal conductance of graphene has an anomalous behavior at low temperatures (below 100 K), with a *T*^3/2^ dependence, while graphite has a *T*^5/2^ dependence, carbon nanotubes have an expected linear temperature dependence and two-dimensional acoustic phonon gases have a T^2^ behavior^12^. The reason for this unexpected behavior for graphene lies in one of the acoustic phonon modes of vibrations, that has a quadratic relationship with the frequency, instead of a linear dependency - as the other two modes. These relationships will thus be explored here in this section, with further consequences for the adiabatic temperature change due to a strain, i.e., the adiabatic barocaloric effect.

Let us consider therefore three acoustic phonon modes with phonon wave vector **q** (|**q**| = *q*), near the center of the Brillouin zone^12,13^:

1. The transverse acoustic (*TA*) mode with *ω* = *υ*_*T*_*q*;
2. The longitudinal acoustic (*LA*) mode with *ω* = *υ*_*L*_*q*; and
3. The flexural out-of-plane acoustic (*ZA*) mode with *ω* = *αq*^2^.

Above, *υ*_*T*_ = 13.6 × 10^3^ m/s is the sound velocity of the transverse wave, *υ*_*L*_ = 21.3 × 10^3^ m/s is the sound velocity of the longitudinal wave and *α* = 6.2 × 10^−7^ m^2^/s represents the coefficient of the quadratic phonons mode of vibration.

Based on the harmonic approximation, in the similar fashion as for the 3D case^10^, graphene has *A*/(2*π*)^2^ **q**-vectors (i.e. vibrational modes) per unit area in the reciprocal space (where *A* is the sample area). In practice, number of crystalografic sites is very large and the reciprocal space is considered as a continuum. Thus, the sum over *j* is replaced by an integral over **q**:

2 $$\sum _{{\bf{j}}}\to \int \,d{\bf{q}}\equiv \frac{A}{{(2\pi )}^{2}}\,\int \,\int \,{d}^{2}q=\frac{A}{{(2\pi )}^{2}}\,\int \,2\pi qdq,$$

using a single integral as a concise notation. It should be stressed that when we actually come to a definite quadrature in **q**-space, we must include the weight factor *A*/(2*π*)^2^. Further considering the aforementioned dispertion relations for the three acoustic phonon modes *ω*(*q*), we obtain:

3 $$\sum _{{\bf{j}}}\to \int \,\frac{A}{2\pi }\,(\frac{\omega }{{\upsilon }_{T}^{2}}+\frac{\omega }{{\upsilon }_{L}^{2}}+\frac{1}{2\alpha })\,d\omega .$$

From this result, it is easy to change the summation of equation 1 to an integral, that reads as:

$$F(T)={\varepsilon }_{0}+\tfrac{A{k}_{B}T}{2\pi }\,(\tfrac{1}{{\upsilon }_{T}^{2}}+\tfrac{1}{{\upsilon }_{L}^{2}})\,{\int }_{0}^{\infty }\,\mathrm{ln}\,(1-{e}^{-\hslash \omega /{k}_{B}T})\,\omega d\omega +\tfrac{A{k}_{B}T}{2\pi }\tfrac{1}{2\alpha }\,{\int }_{0}^{\infty }\,\mathrm{ln}\,(1-{e}^{-\hslash \omega /{k}_{B}T})\,d\omega ,$$

and, due to its rapid convergence for small *k*_*B*_*T* values, it can be taken from 0 to ∞. After a change of variables of the kind $z=\hslash \omega /{k}_{B}T$, the final result for the free energy is:

4 $$F(T)={\varepsilon }_{0}-\frac{A{k}_{B}^{3}{T}^{3}}{4\pi {\hslash }^{2}}\,(\frac{1}{{\upsilon }_{T}^{2}}+\frac{1}{{\upsilon }_{L}^{2}})\,{\rm{\Gamma }}(3)\zeta (3)-\frac{A{k}_{B}^{2}{T}^{2}}{2\pi \hslash }\frac{1}{2\alpha }\frac{{\pi }^{2}}{6}$$

where Γ(…) and *ζ*(…) are, respectively, the Gamma and Riemann Zeta functions.

The entropy can be derived from *S* = −∂*F*/∂*T* and therefore the *lattice entropy per graphene area S*_*la*_(*T*) reads as:

5 $${S}_{la}(T)={k}_{B}\frac{3{k}_{B}^{2}{T}^{2}}{4\pi {\hslash }^{2}}\,(\frac{1}{{\upsilon }_{T}^{2}}+\frac{1}{{\upsilon }_{L}^{2}})\,{\rm{\Gamma }}(3)\zeta (3)+{k}_{B}\,\frac{\pi {k}_{B}T}{12\hslash \alpha }$$

It is worth to note that the linear term on the temperature depends on the *α* term, i.e., on the anomalous out-of-plane acoustic quadratic mode; while the quadratic temperature term depends on the linear transverse (*TA*) and longitudinal acoustic (*LA*) modes. These dependencies have further implications on the adiabatic barocaloric effect of this material.

### Strain entropy

The present subsection deals with the *strain entropy per graphene area S*_*s*_(*T*, *B*_*s*_), that, in other words, is a consequence of a pseudo-magnetic field due to strain^5–8^. It has been described in our previous work^3^ and reads as:

6 $${S}_{s}(T,{B}_{s})={S}_{0}(T)+{S}_{osc}(T,{B}_{s}),$$

where

7 $${S}_{0}(T)={N}_{0}{k}_{B}\frac{2{\pi }^{2}{k}_{B}T}{3{\epsilon }_{F}^{0}}$$

is the strain-independent term^14^, ${\epsilon }_{F}^{0}=\hslash {\upsilon }_{F}\sqrt{\pi {N}_{0}}$ is the zero-strain Fermi energy, *N*_0_ = 10^16^ m^−2^ is the density of charge carriers and *υ*_*F*_ = 10^6^ m/s is the Fermi velocity. The strain-dependent term oscillates as a function of the reciprocal pseudo-magnetic field 1/*B*_*s*_, and it has been comprehensively described recently^3^. It reads as:

8 $${S}_{osc}(T,{B}_{s})={N}_{0}{k}_{B}\,\sum _{\eta }\,\frac{1}{m}\,\sum _{k=1}^{\infty }\,\frac{1}{k}\,\cos \,(\pi km)\,{\mathscr{T}}\,({x}_{k})$$

where

9 $$m=\frac{{N}_{0}{\varphi }_{0}}{|\eta {B}_{s}|},$$

${\varphi }_{0}=\pi \hslash /e=2.06\times {10}^{-15}$ Tm^2^ is the magnetic flux quantum and *η* = ±1 is the valley index and is associated to the Dirac points (*K*_+1_ and *K*_−1_) in the first Brilloiun zone. The thermal dependence of the strain entropy is contained in the function:

10 $${\mathscr{T}}\,({x}_{k})=\frac{{x}_{k}L\,({x}_{k})}{\sinh \,{x}_{k}},\,{\rm{where}}\,L({x}_{k})=\,\coth \,({x}_{k})-\frac{1}{{x}_{k}}$$

is the Langevin function. Above,

11 $${x}_{k}=kx=k\frac{m}{{N}_{0}}2\pi \frac{{\epsilon }_{F}{k}_{B}T}{{\hslash }^{2}{\upsilon }_{F}^{2}},\,{\rm{where}}\,{\epsilon }_{F}=\hslash {\upsilon }_{F}\sqrt{{(\frac{\bar{\gamma }{B}_{s}}{\hslash {\upsilon }_{F}})}^{2}+\pi {N}_{0}}$$

is the strain-dependent Fermi level and $\bar{\gamma }\approx 1.7{\mu }_{B}$. Note that both *m* and *x* are dimensionles.

## Discussion

The evaluation of the barocaloric effect under adiabatic condition requires the total entropy^15^ to be written as a function of the strain and lattice entropies. Thus:

12 $${S}_{tot}(T,{B}_{s})={S}_{la}(T)+{S}_{s}(T,{B}_{s})$$

where these two terms were presented, respectively, on equations 5 and 6. It is a different situation from our previous contribution on reference^3^, in which the isothermal barocaloric effect was discussed. For that case, the field-independent contribution to the total entropy, i.e., the lattice term, has been canceled when the isothermal entropy change was calculated. This is the reason the lattice term has not been explicitly considered in reference^3^.

However, numerical evaluation of those terms on equation 5, for $T\lessapprox \,100$, clearly shows that the quadratic term on temperature is much smaller than the linear one: at 1 K (100 K) it is three (one) order of magnitude smaller. As a consequence, the linear modes of phonon vibrations have no effects for the lattice entropy in comparison to the anomalous quadratic mode. Thus, the total entropy can thus be written as:

13 $${S}_{tot}(T,{B}_{s})={N}_{0}{k}_{B}\frac{2{\pi }^{2}{k}_{B}T}{3{\epsilon }_{F}^{0}}+{k}_{B}\frac{\pi {k}_{B}T}{12\hslash \alpha }+{S}_{osc}(T,{B}_{s})$$

A simple numerical comparison of the first and second terms above shows that the second term, related to the lattice entropy, is three orders of magnitude bigger and therefore we can disregard the first term. The final total entropy we must use to obtain the adiabatic temperature change due to strain is therefore:

14 $${S}_{tot}(T,{B}_{s})={k}_{B}\frac{\pi {k}_{B}T}{12\hslash \alpha }+{N}_{0}{k}_{B}\,\sum _{\eta }\,\frac{1}{m}\,\sum _{k=1}^{\infty }\,\frac{1}{k}\,\cos \,(\pi km)\,{\mathscr{T}}\,({x}_{k})$$

Note there is one term exclusively dependent on parameters related to the lattice (*α*); and other term exclusively dependent on parameters related to the strain energy (*B*_*s*_).

In order to obtain the adiabatic temperature change, let us consider a *S*_*tot*_(*T*, *B*_*s*_) diagram, with two entropy curves: one with and other without pseudo-magnetic field *B*_*s*_, i.e., strain. The adiabatic temperature change is a measurement of the ‘distance’ (in units of Kelvin), between these two curves, for a constant value of entropy. In other words, the condition:

15 $${S}_{tot}({T}_{0},0)={S}_{tot}({T}_{{B}_{s}},{B}_{s})$$

applies, where *T*_0_ and ${T}_{{B}_{s}}$ are, respectively, the temperature of the graphene under zero and non-zero pseudo-magnetic field, i.e., under zero- and finite strain. From this condition, the final temperature of the body ${T}_{{B}_{s}}$ after the strain can be written; and the adiabatic temperature change ${\rm{\Delta }}T({T}_{0},{B}_{s})={T}_{{B}_{s}}-{T}_{0}$ formally found. This last quantity is the amount of temperature the material changes (either cooling or heating), due to the strain. This is one way to measure the *barocaloric effect*. The other way is considering the isothermal entropy change Δ*S*(*T*, *B*_*s*_) = *S*_*s*_(*T*, *B*_*s*_) − *S*_*s*_(*T*, 0) and has already been discussed^3^.

**Figure 1:**
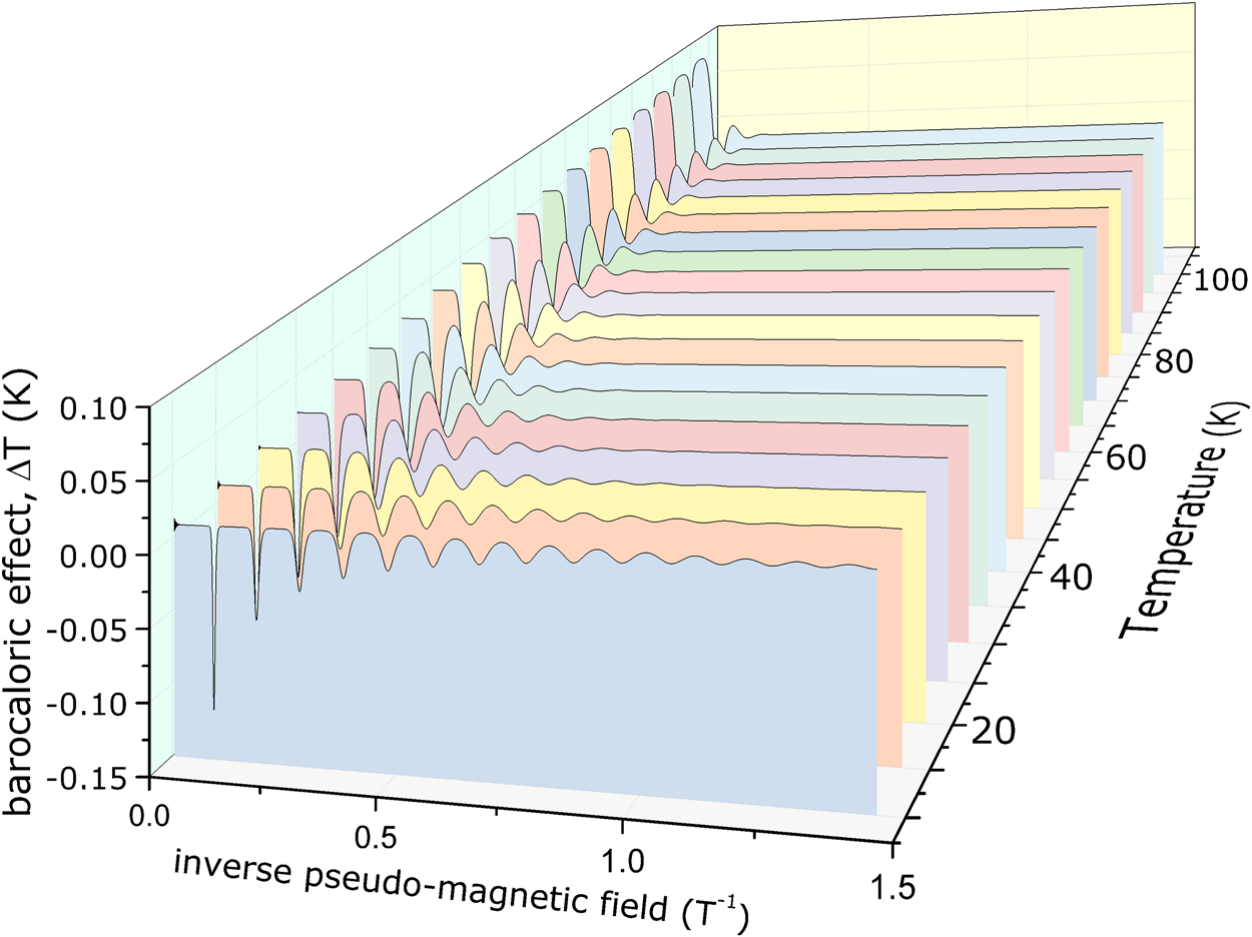
Adiabatic temperature change as a function of the inverse pseudo-magnetic field. This caloric effect, as well as the oscillations, emerge due to the strain on the graphene sheet and the physical mechanism behind this phenomena is the anomalous quadratic phonons mode of vibration. The first curve represents the BCE at 10 K. The strain is given in Tesla, due to the pseudo-magnetic field; however, the strain energy^3^ is $\bar{\gamma }{B}_{s}$, where $\bar{\gamma }=9.788\times {10}^{-5}$ eV/T. The inverse pseudo-magnetic field shown above can be expressed in terms of the reciprocal energy (eV^−1^), after a simple multiplication of that scale by $1/\bar{\gamma }$.

Our interest is therefore to find ${T}_{{B}_{s}}({T}_{0})$, i.e., the final temperature of the graphene sheet after a certain strain, starting from a given initial temperature *T*_0_. The adiabatic condition (equation 15), after some standard calculation, provides the inverse function ${T}_{0}({T}_{{B}_{s}})$, that reads as:

16 $${T}_{0}={T}_{{B}_{s}}+(24\pi \frac{{N}_{0}\hslash \alpha }{{\epsilon }_{F}^{0}})\,(\frac{{\epsilon }_{F}}{{\epsilon }_{F}^{0}}){T}_{{B}_{s}}\,\sum _{\eta }\,\sum _{k=1}^{\infty }\,\cos \,(\pi km)\frac{L(k{x}_{{B}_{s}})}{\sinh \,(k{x}_{{B}_{s}})},$$

where ${x}_{{B}_{s}}=x({T}_{{B}_{s}})$. It is worth to note that it is not possible to invert this equation to obtain ${T}_{{B}_{s}}({T}_{0})$ and therefore it must be found self-consistently, for a given *T*_0_. Once ${T}_{{B}_{s}}$ has been found numerically from the above equation, it is possible to obtain the final result: the adiabatic temperature change ${\rm{\Delta }}T({T}_{0},{B}_{s})={T}_{{B}_{s}}-{T}_{0}$. This result is presented on Fig. 1, for several values of temperature, from 1 K up to 100 K.

The core of this effort is the adiabatic temperature change due to strain, that only exists because of the second term of equation 16, that, on its turn, is ruled by the anomalous acoustic phonons mode of vibration by means of the parameter *α*. Thus, further attention must be given to this parameter. Positive values of *α* reveal that should exist critical points at which *ω* is near a local minimum and the sound velocity vanishes. Moreover, it largely depends on the local second derivative of *ω* with respect to the *q*, i.e., $\alpha =(1/2)\,({\partial }^{2}\omega /\partial {q}^{2})$; therein *ω* band could be derived from numerical simulation, experiment, and other methods^16–18^. On the other side, the *α*-term has been pointed out to be related to the bending rigidity, the atomic density, and so forth^19^, which would pave some possible ways to modulate the *α*-dependence physical effects. As discussed above, the existence of ZA modes leads to some interesting phenomena on graphene, including the barocaloric effect.

Also remarkable are the quantum oscillations; a clear indication that the Landau Levels exist for this system and, consequently, the pseudo-magnetic field. These oscillations are notorious for lower values of temperature (c.a. 10 K). It is worth to note that the adiabatic barocaloric effect is either positive or negative depending on the value of pseudo-magnetic field *B*_*s*_. In other words, for a certain value of strain energy, the graphene sheet heats up; and for other values of strain energy, the material cools down. It is a consequence of the oscillatory adiabatic barocaloric effect: a truly unique quantum effect, ruled by the anomalous quadratic phonons mode of vibration.

## Conclusion

The adiabatic temperature change is one way to obtain the caloric effect and it is obtained after a change on an external parameter as magnetic or electric fields, pressure and even rotation of the material. Graphene, on its turn, has the marvelous property to produce a local pseudo-magnetic field as a consequence of strain - and this pseudo-magnetic field can be as large as 300 Tesla^8^. This pseudo-magnetic field is therefore the excitation for the *barocaloric effect*; and the present effort describes the physical mechanisms that govern the effect.

In order to obtain the adiabatic temperature change, the total entropy of the graphene was written, including the lattice and the strain terms. The lattice entropy contains two terms: one linear with the temperature and dependent on the anomalous quadratic phonon mode; and other term quadratic with the temperature and dependent on the linear transverse and longitudinal phonon modes. The temperature-quadratic term, for $T\lessapprox \,100$, is much smaller than the linear term and has been disregarded. As a consequence, the adiabatic *barocaloric effect*, is completely ruled by the existence of the anomalous out-of-plane phonon mode, leading therefore to a unique and interesting caloric effect on graphene. It is also possible to cite oscillatory effect, in which the graphene is able to increase or decrease its temperature due to the strain energy.

## Methods

Caloric effects can be seen from an adiabatic temperature change or an isothermal entropy change. The isothermal entropy change, i.e., the isothermal *barocaloric effect* was computed previously on reference^3^, and to achieve that result only the field-dependent terms to the total entropy were needed, since field-independent terms are canceled upon evaluation of the entropy change due to a field change. Considering equation 12:

17 $${S}_{tot}(T,{B}_{s})={S}_{la}(T)+{S}_{s}(T,{B}_{s}),$$

it is possible to write the isothermal entropy change:

18 $$\begin{array}{rcl}{\rm{\Delta }}{S}_{tot}(T,{\rm{\Delta }}{B}_{s}) & = & \{{S}_{la}(T)+{S}_{s}(T,{B}_{s})\}-\{{S}_{la}(T)+{S}_{s}(T,0)\}\end{array}$$

19 $$\begin{array}{rcl} & = & {S}_{s}(T,{B}_{s})-{S}_{s}(T,0),\end{array}$$

that, in fact, does not depend on the lattice term.

On the other hand, for the adiabatic temperature change, i.e., the adiabatic *barocaloric effect*, the central issue of the present effort, the field-independent term to the total entropy (the lattice entropy), plays a key role. In order to understand the mechanism, let us consider a simple spin-lattice system: an application of a magnetic field aligns the spins, decreasing its magnetic entropy. To maintain the total entropy constant, the lattice entropy must increase; and thus the consequence is an increasing of the temperature of the system. For this case, the adiabatic condition shall be applied (equation 15):

20 $${S}_{tot}({T}_{0},0)={S}_{tot}({T}_{{B}_{s}},{B}_{s}),$$

and, from this, the adiabatic temperature change Δ*T* = *T*_*B*_ − *T*_0_ can be obtained (either analytically or numerically, depending on the system).
